# 1934. Association between Receipt of COVID-19, Influenza, and Pneumococcal Vaccination

**DOI:** 10.1093/ofid/ofac492.1561

**Published:** 2022-12-15

**Authors:** Chris Choi, Ashley Tippett, Luis W Salazar, Laila Hussaini, Olivia D Reese, Khalel De Castro, Grace Taylor, Meg Taylor, Caroline R Ciric, Laura A Puzniak, Robin Hubler, Srinivas Valluri, Benjamin Lopman, Satoshi Kamidani, Christina A Rostad, John M McLaughlin, Evan J Anderson

**Affiliations:** Emory University, Atlanta, Georgia; Emory University, Atlanta, Georgia; Emory University, Atlanta, Georgia; Emory Univeristy, Atlanta, Georgia; Emory University, Atlanta, Georgia; Emory University, Atlanta, Georgia; Emory University, Atlanta, Georgia; Emory University, Atlanta, Georgia; Emory University, Atlanta, Georgia; Pfizer Inc., Collegeville, Pennsylvania; Pfizer Inc., Collegeville, Pennsylvania; Pfizer Inc, New York, New York; Rollins School of Public Health | Emory University, Atlanta, Georgia; Emory University School of Medicine and Children's Healthcare of Atlanta, Atlanta, Georgia; Emory University School of Medicine and Children's Healthcare of Atlanta, Atlanta, Georgia; Pfizer, Collegeville, Pennsylvania; Emory University School of Medicine, Atlanta, Georgia

## Abstract

**Background:**

Whether receipt of COVID-19 vaccine associates with receipt of other routinely-recommended adult vaccines such as, influenza and pneumococcal vaccines is not well described. We evaluated this relationship in a population of adults who were hospitalized for acute respiratory infection (ARI).

**Figure:**
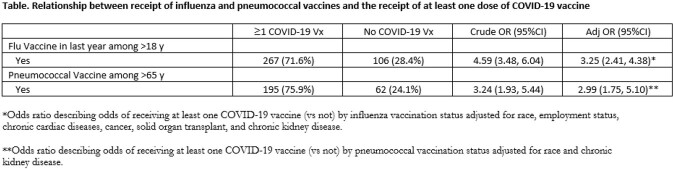

**Methods:**

We enrolled adults (≥ 18 years of age) who were hospitalized at Emory University Hospital and Emory University Hospital Midtown with symptoms consistent with ARI. Participants were interviewed and medical records abstracted to gather demographic information, including social behaviors during the pandemic, medical history, and prior vaccination history (i.e., COVID-19, influenza, and pneumococcal). Using two separate logistic regression analyses, we determined the association between i) receipt of influenza vaccine in the prior year among adults ≥ 18 years and ii) receipt of any pneumococcal vaccine in the prior 5 years among adults ≥ 65 years on the receipt of at least one COVID-19 vaccine≥ 14 days prior to admission. Adjusted models included demographic information (e.g., age, sex, race/ethnicity, employment status), social behaviors, and history of chronic medical conditions.

**Results:**

Overall, 1056 participants were enrolled and had vaccination records available. Of whom, 509/1056 (48.2%) had received at least one dose of COVID-19 vaccine. Adults ≥ 18 years who received influenza vaccine were more likely to have received ≥1 dose of COVID-19 vaccine compared to those who did not (267/373 [71.6%] vs 242/683 [35.4%] P=< .0001; adjusted odds ratio [OR]: 3.3 [95%CI: 2.4, 4.4]). Similarly, adults ≥65 years who received pneumococcal vaccine were more likely to have received ≥ 1 dose of COVID-19 vaccine compared to those who did not (195/257 [75.9%] vs 41/84 [48.8%] P=< .0001; adjusted odds ratio [OR]: 3.0 [95%CI: 1.8, 5.1]).

**Conclusion:**

In this study of adults hospitalized for ARI, receipt of influenza and pneumococcal vaccination strongly correlated with receipt of COVID-19 vaccination. Continued efforts are needed to reach adults who remain hesitant to not only receive COVID-19 vaccines, but also other vaccines that lessen the burden of respiratory illness.

**Disclosures:**

**Laura A. Puzniak, PhD. MPH**, Merck & Co., Inc.: Stocks/Bonds|Pfizer, Inc.: Stocks/Bonds **Robin Hubler, MS**, Pfizer Inc.: Employee|Pfizer Inc.: Stocks/Bonds **Srinivas Valluri, PhD**, Pfizer: Employee|Pfizer: Stocks/Bonds **Benjamin Lopman, PhD**, Epidemiological Research and Methods, LLC: Advisor/Consultant **Satoshi Kamidani, MD**, NIH: His institution (Emory University) receives funds from Pfizer for his work as a co-investigator on clinical trials of Pfizer COVID-19 vaccine.|Pfizer: His institution (Emory University) receives funds from Pfizer for his work as a co-investigator on clinical trials of Pfizer COVID-19 vaccine. **Christina A. Rostad, MD**, BioFire Inc, GSK, MedImmune, Micron, Merck, Novavax, PaxVax, Pfizer, Regeneron, Sanofi-Pasteur.: Grant/Research Support|Meissa Vaccines, Inc.: Co-inventor of RSV vaccine technology licensed to Meissa Vaccines, Inc.|NIH (Funding from NIH to conduct clinical trials of Moderna and Janssen COVID-19 vaccines): Grant/Research Support **John M. McLaughlin, PhD**, Pfizer: Employee|Pfizer: Stocks/Bonds **Evan J. Anderson, MD**, GSK: Advisor/Consultant|GSK: Grant/Research Support|Janssen: Advisor/Consultant|Janssen: Grant/Research Support|Kentucky Bioprocessing, Inc: Data Safety Monitoring Board|MedImmune: Grant/Research Support|Medscape: Advisor/Consultant|Merck: Grant/Research Support|Micron: Grant/Research Support|NIH: Funding from NIH to conduct clinical trials of Moderna and Janssen COVID-19 vaccines|PaxVax: Grant/Research Support|Pfizer: Advisor/Consultant|Pfizer: Grant/Research Support|Regeneron: Grant/Research Support|Sanofi Pasteur: Advisor/Consultant|Sanofi Pasteur: Grant/Research Support|Sanofi Pasteur: Data Adjudication and Data Safety Monitoring Boards|WCG and ACI Clinical: Data Adjudication Board.

**Figure:** *Odds ratio describing odds of receiving at least one COVID-19 vaccine (vs not) by influenza vaccination status adjusted for race, employment status, chronic cardiac diseases, cancer, solid organ transplant, and chronic kidney disease.

**Odds ratio describing odds of receiving at least one COVID-19 vaccine (vs not) by pneumococcal vaccination status adjusted for race and chronic kidney disease.

